# Subjective Norm Rather Than Social Norm in the Induced-Hypocrisy Paradigm: A Test in the Context of School Bullying Victim Support

**DOI:** 10.5334/irsp.776

**Published:** 2023-10-25

**Authors:** Maxime Mauduy, Jessica Mange

**Affiliations:** 1 Université Paris Cité, Laboratoire de Psychologie Sociale : contextes et régulation (LPS, UR 4471), Institut de Psychologie, Boulogne-Billancourt, France; 2 Université de Caen Normandie, Laboratoire de Psychologie Caen Normandie (LPCN, EA 7452), France

**Keywords:** induced-hypocrisy paradigm, subjective norm, social norm, school bullying, victim-defending intention

## Abstract

The induced-hypocrisy paradigm is an effective two-step procedure—normative-salience step and then transgressions-salience step—for encouraging normative behaviors. In the context of promoting school bullying victim support among witnesses, this study tests whether the activation of a subjective norm rather than a social norm as traditionally practiced in the hypocrisy procedure can enhance the hypocrisy effect. Middle school students (*N* = 191) were assigned to either the control, social-norm-hypocrisy, or subjective-norm-hypocrisy conditions. Victim-defending intentions were measured immediately and one month later. The results showed a significant increase, ranging from control, then social-norm-hypocrisy, to subjective-norm-hypocrisy conditions, in students’ victim-defending intentions. These results extend the scope of induced hypocrisy and contribute to progress in investigating processes underlying the hypocrisy effect.

School bullying is considered a major public health issue (Salmivalli, 2014). Researchers focus their attention on school bullying witnesses to significantly prevent school bullying situations. Though their pro-victim reactions could put an end to bullying, the large majority of witnesses remain passive (Salmivalli, 2014). Consequently, identifying prevention strategies to encourage witnesses’ pro-victim reactions is critical. Besides, a recent preliminary study seems to show the effectiveness of induced hypocrisy in promoting victim support (Mauduy, Bagneux & Sénémeaud, 2023). In this two-step procedure, people promote a normative behavior (the ‘normative-salience step’; e.g., bullying is socially disapproved) and then recall their own past failures to comply with it (the ‘transgressions-salience step’; e.g., recalling their own passive reactions to bullying). Making this inconsistency salient generates the hypocrisy effect (Stone & Fernandez, 2008), leading people to adopt behaviors in accordance with the norm. The present research aims to replicate the induced-hypocrisy effect on witnesses’ victim-defending intentions but also particularly intends to suggest a way to enhance it based on recent results showing the importance of the normative-salience step.

When the induced-hypocrisy paradigm (IHP) was established, the main explanation of its effect was the self-consistency theory (Aronson, 1999). According to it, the hypocrisy effect is only driven to restore the individual self-concept threatened by the recall of past transgressions. However, a recent explanation, namely the deviation-from-norm approach (Liégeois et al., 2017), attaches a more central role to the normative-salience step. The function of this step would be to make the social norm related to the given behavior salient (Stone & Fernandez, 2008). The behavioral change in the IHP would therefore help reduce the gap between norm and behavior. Some recent results support this approach by showing that the inconsistency between a social norm and transgressions generates the hypocrisy effect (e.g., Mauduy et al., 2022) and the normative salience strength (weak vs. strong) moderated the hypocrisy effect size (Mauduy et al., 2023). Consequently, if the social norm plays a determinant role in the hypocrisy effect, then the characteristics of the activated social norm could influence the hypocrisy effect.

Traditionally, in the normative-salience step, a general social norm is made salient whereby such behavior is socially approved or not in our society (Liégeois et al., 2017). Thus, the activated norm is not specific to a particular situation or group. However, the subjective norm refers to the behavior expected of an individual from significant others in one’s social environment (Chung & Rimal, 2016). It is a situationally and specific social norm that has a greater influence on behavior than the general social norm (Reese et al., 2014). In school settings, most pupils consider adults (i.e., teachers and educational staff) as significant persons (Veenstra et al., 2010). Moreover, the subjective anti-bullying norm (Mauduy, Bagneux & Sénémeaud, 2021) and more specifically the adult-related subjective norm influences witnesses’ reactions (Kubiszewski et al., 2019). Thus, we expected that within the IHP, the activation of an adult-related subjective anti-bullying norm, rather than a usually general social norm should increase the hypocrisy effect.

## Method

### Sample and study design

A total of 191 ninth-grade students (*M*_age_ = 14.41, *SD* = 0.55; 53.4% girls) from two middle schools in Normandy (France) participated in the study on a voluntary basis. This sample size allowed us to achieve a sufficient statistical power of .82 (see supplemental material on OSF at https://osf.io/w9rz7/?view_only=1fb171257201401193458eaae2db35ae).

A quasi-experimental longitudinal trial was conducted over a 1-month period. Students were approached collectively in classrooms and were balanced across three conditions: control (*n* = 46; two classes), social-norm-hypocrisy (*n* = 69; three classes), and subjective-norm-hypocrisy (*n* = 76; three classes). Gender and age are equally distributed across the three conditions (see Supplemental Table 1). At the end of the intervention, the students’ intentions to passive and pro-victim reactions in school bullying situations were immediately measured (time 1) and then again 1-month later (time 2). Finally, the students were debriefed to inform them of the study purpose and were asked to address any questions. In the control condition, the students were directly asked to complete the dependent variables for two reasons. First, it allowed us to observe the possible benefits of our intervention with respect to the existing situation in the schools. Second, the normative-salience-only condition does not differ significantly from a control condition (for a meta-analysis, see Priolo et al., 2019), and only the articulation of the two IHP steps leads to the hypocrisy effect (e.g., Aronson et al., 1991).

### Ethics

The survey was conducted in full agreement with the ethical standards set by the Psychology Department that follow the American Psychological Association’s Ethical Principles of Psychologists and the Code of Conduct (APA, 2017) for the ethical treatment of human participants. The protocol was submitted to the school departments of the Normandy region as well as to the school headmasters and to the teachers of each class involved in the study. Prior to data collection, individual consent for participation and active parental consent were requested and obtained.

### Materials

#### Induced-hypocrisy intervention

For students in the *social-norm-hypocrisy* condition, the same hypocrisy intervention as that used by Mauduy, Bagneux, and Sénémeaud (2023) was performed. First, students performed the normative-salience step aiming to make the social anti-bullying norm salient (Liégeois et al., 2017). Concretely, they were asked to list arguments explaining the reasons why school bullying is socially disapproved in our society. Second, the students performed the transgressions-salience step. Concretely, they were invited to individually and anonymously complete a questionnaire making six own past passive behaviors in witness situations salient.

Students in the *subjective-norm-hypocrisy* condition followed the same hypocrisy intervention as the students in the *social-norm-hypocrisy* condition, except for the normative-salience step which was modified to make the adult-related subjective anti-bullying norm salient. The students were asked to list arguments showing that school bullying is frowned upon by significant others, namely adults in a school setting (teachers and educational staff).

#### School bullying victim-defending behavioral intentions

A questionnaire, adapted from Mauduy, Bagneux, and Sénémeaud (2021), was created to measure defending behavioral intentions. In this questionnaire (see supplemental material), the students were invited to indicate how they could react in five situations in which they witnessed school bullying by answering four items (on a five-point scale: (1) *Never*–(5) *Always*) each time and for each situation. One item served to measure passive behavioral intentions and, based on the three victim-defending reactions (i.e., *supporting* – comforting and supporting him/her – *opposing* – intervening in opposition to the aggressor – and *alerting* – seeking the help of adults – Salmivalli, 2014), three other items served to measure pro-victim behavioral intentions. Analyses showed good reliabilities for the five items of passive (Cronbach’s α = .80) and 15 items of pro-victim (Cronbach’s α = .88) behavioral intentions.

### Data analysis and hypothesis

A linear mixed model with the bootstrapping method in Jamovi (Gallucci, 2019) was constructed for behavioral intentions, with the conditions as between-participants fixed component, the type of behaviors (i.e., passive and pro-victim) and the time of measures (T1 and T2) as within-participants fixed components, and the participant variable as a random component. The passive intentions score was reversed, hence indicating non-passive intentions to match the direction of the pro-victim intentions score. Z-scores of these two measures were included in the model to facilitate their comparison. As previous studies have shown that students’ gender influences witnesses responses to school bullying situations (e.g., Gini et al., 2015), we included in the model gender as a covariate (Girl: –0.5; Boy: 0.5; see supplemental material for model results without gender as a covariate). We expected a main effect of our conditions on students’ defending intentions. More precisely, a significant increase, ranging from control, then social-norm-hypocrisy, to subjective-norm-hypocrisy conditions, in students’ victim-defending intentions would be observed. To test this specific hypothesis, we used the contrast method as recommended by many authors (Judd et al., 2017) and more particularly the polynomial contrasts. To conclude that the data were consistent with our hypothesis, the linear contrast (i.e., control, then social-norm-hypocrisy, and then subjective-norm-hypocrisy conditions) had to explain a significant part of the variance of the outcomes while the quadratic one had to be non-significant. Preliminary analyses were carried out to verify that the number of recalled transgressions did not differ between our two hypocrisy conditions, that gender did not interact with our variables, and that it was not necessary to account for the random factor ‘class’ in our model (see supplemental material).

## Results

Descriptive statistics are displayed in Table 1.

**Table 1:** Means and standard deviations for study measures across time and conditions.


MEASURES	PASSIVE BEHAVIORAL INTENTIONS		PRO-VICTIM BEHAVIORAL INTENTIONS	
		
CONDITIONS	T1	T2	T1	T2

Control	2.23 (0.80)	2.04 (0.68)	3.31 (0.89)	3.34 (0.76)

Social-norm-hypocrisy	2.04 (0.91)	1.98 (0.77)	3.49 (0.78)	3.44 (0.78)

Subjective-norm-hypocrisy	1.78 (0.63)	1.90 (0.76)	3.68 (0.62)	3.66 (0.65)


*Note*: *N* = 191. Measures at T1 were assessed immediately after the intervention. T2 were assessed one month after T1.

**Figure 1:**
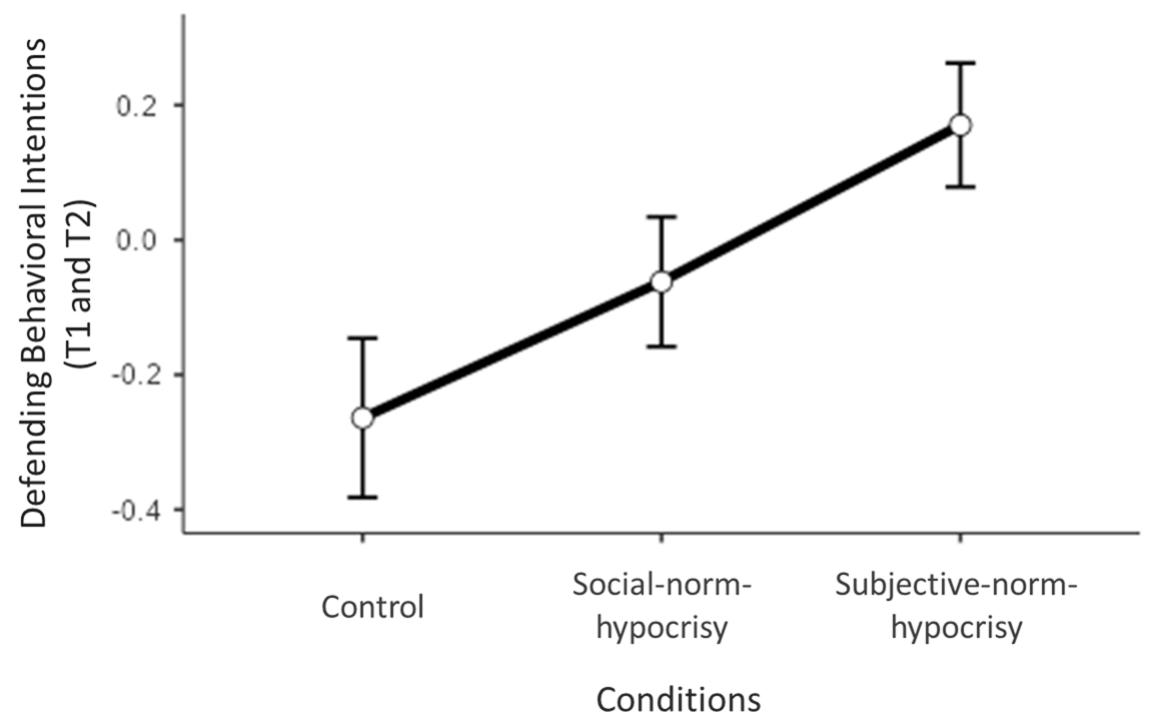
Defending behavioral intentions (at T1 and T2) across the conditions. *Note*: *N* = 191. Data show means and *SE*. Defending behavioral intentions are a *z*-score combining the scores at T1 and T2 for non-passive and pro-victim intentions.

Overall, the linear mixed model explained 10.1% of the students’ defending behavioral intentions. First, the results showed a significant main effect of our conditions, *F*(2,187) = 4.34, *p* = .014. More precisely, the linear contrast (*Estimate* = 0.31, *SE* = 0.11, *p* = .004) was significant on behavioral intentions while the quadratic contrast was not (*Estimate* = 0.01, *SE* = 0.10, *p* = .9). These results support our hypothesis since they show a significant and positive trend of control (*M* = –0.26, *SE* = 0.12), social-norm-hypocrisy (*M* = –0.06, *SE* = 0.10), and subjective-norm-hypocrisy (*M* = 0.17, *SE* = 0.09) conditions on students’ defending intentions (see Figure 1). Moreover, the results did not show any significant interaction between the conditions and type of behaviors, *F*(2,564) = 0.30, *p* = .74, as well as between conditions and times, *F*(2,564) = 2.02, *p* = .13. The latter result suggests that our conditions influenced students’ intentions at T1 and T2, which is supported by the significant effects of the linear contrast both at T1 (*Estimate* = 0.39, *SE* = 0.11, *p* < .001) and T2 (*Estimate* = 0.23, *SE* = 0.11, *p* = .04). Finally, gender was significant (*Estimate* = –0.53, *SE* = 0.12, *p* < .001) and the conditions × type of behaviors × times interaction was not significant, *F*(2,564) = 1.58, *p* = .20).

## Discussion

The current study (1) replicates the induced-hypocrisy effect in the context of school bullying victim support and (2) shows that using the subjective norm in the induced-hypocrisy procedure increases the hypocrisy effect obtained from a social norm. These findings have both practical and theoretical implications.

First, the IHP may prove to be a new effective and relevant strategy to be directly mobilized by the educational staff and especially teachers to prevent school bullying. Indeed, teachers and their relationship with students play an important role in bullying (Lambe et al., 2019). Therefore, an induced hypocrisy intervention targeting the teacher-related subjective anti-bullying norm that would be delivered directly in the classroom by the teachers themselves could be relevant and particularly effective. This could supplement prevention programs that target the role of witnesses, such as the Kiva program. Indeed, this program aims to develop interventions, in the form of teacher-delivered lessons, that intend to strengthen students’ normative anti-bullying attitudes and beliefs and sense of self-efficacy in defending reactions (Salmivalli, 2014). Yet, none of the proposed interventions target the inconsistency of witnesses who have a psychological profile against bullying but who demonstrate passive behaviors (i.e., ‘inconsistent’ witnesses, Mauduy, Bagneux & Sénémeaud, 2021). In this regard, induced hypocrisy could be a relevant new strategy to mobilize in this program. However, for practical purposes, we emphasize that various ways exist to reduce cognitive dissonance. It therefore seems important, until proven otherwise, to orient the students’ reduction strategy after the hypocrisy procedure to ensure that it supports the expected behavior, and not the opposite (e.g., morally disengage from the situation).

Second, our results contribute to investigate the explanatory processes underlying the hypocrisy effect. First, the deviation-from-norm approach (Liégeois et al., 2017; Mauduy et al., 2022) considers the importance of social norms. Consistent with a recent meta-analysis (Mauduy et al., 2023) showing that the normative-salience strength moderated the hypocrisy effect, our results could be explained in terms of normative level of the normative-salience step, with a stronger normative salience induced by the subjective norm, compared to the social norm. However, our results could also be explained by a greater self-threat, as predicted by self-consistency theory (Aronson, 1999). Indeed, inconsistency with a subjective norm would be more important and threatening to the self than inconsistency with a social norm, since it would involve important people and/or people closer to the self. As people follow social norms to avoid social sanctions and more specifically follow subjective ones to maintain interpersonal harmony (Chung & Rimal, 2016), in other words, to satisfy their need to belong, the later could thus be the part of the self which is more involved in the inconsistency with a subjective norm than social one in induced-hypocrisy situations.

In conclusion, this study shows the role of the normative-salience step in obtaining the hypocrisy effect and the relevance of using IHP in school bullying. A first limitation concerns our study design, which includes neither a normative-salience step alone, nor a transgression-salience step alone. Our results could simply be guided by the manipulation of normative salience. However, this would run counter to 30 years of IHP research since it suggests that the transgressions-salience step played no role in our results. Our results could also be only due to the transgressions salience. However, this interpretation would not explain the difference between subjective-hypocrisy and social-hypocrisy conditions. Anyway, it would be interesting for bullying prevention purposes to test the added value of the hypocrisy effect compared to each step. Secondly, replicating this subjective-hypocrisy effect in a different context through laboratory studies could be beneficial. Thirdly, the transgressions (i.e., lack of pro-victim behaviors) were not directly related to the anti-bullying norm, which may have weakened our effects. Accentuate the incoherence—and thus strengthen the hypocrisy effect—by making the pro-victim of school bullying norm salient would be an interesting research perspective. Moreover, working on the recalled transgressions could also reinforce the hypocrisy effect in the bullying context, as the transgressions are passive here, and therefore less engaging than active behaviors. Despite these limitations, our study results invite future research to investigate the psychological processes underlying the influence of social norms in IHP, particularly the role of threat to the need to belong. Continuing to study this issue in the context of school bullying seems especially relevant, given the importance of the need to belong among adolescents.

## Data Accessibility Statement

The dataset is available on OSF at https://osf.io/w9rz7/?view_only=1fb171257201401193458eaae2db35ae.
